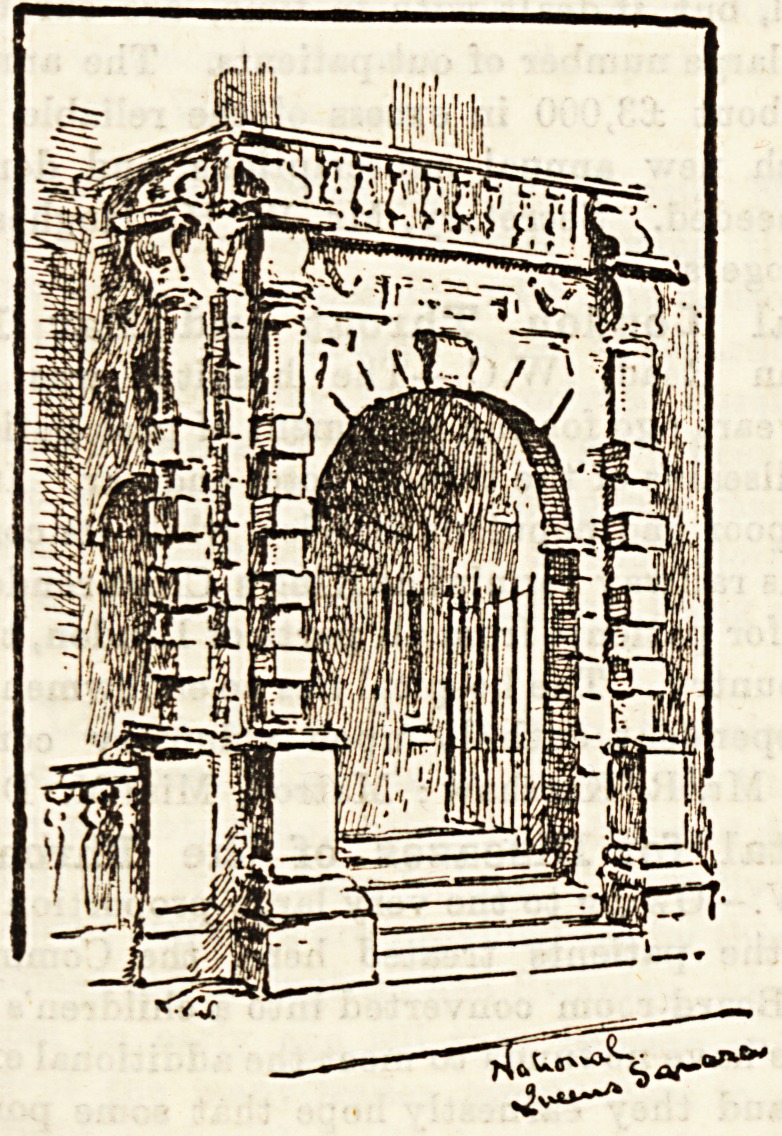# Epilepsy and Paralysis

**Published:** 1892-12-24

**Authors:** 


### EPILEPSY AND PARALYSIS.

**Hospital for Epilepsy and Paralysis,** Portland Terrace, Regent's Park, N.W.—At this institution, in order to promote providence and to minimise abuse, patients are encouraged to pay what they can afford. The payments from these patients does not, however, nearly cover the cost of the treatment, while those unable to pay receive free relief. The hospital being unendowed, there necessarily remains a large sum to be collected each year, and the committee, therefore, are appealing for at least £1,000 in new annual subscriptions. Secretary, Mr. Howgrave Graham; Matron, Miss Ridley.

**National Hospital for Paralised and Epileptic,** Queen Square, W.C.—This hospital relieves a class of sufferers terribly afflicted, large in numbers, and unfitted for general hospitals for whom little provision is made in any other institution. It provides 170 beds and a spacious out-patients' department, while a surgical wing is also in course of erection. It is manifest how insufficient 200 beds must be to meet the large demands made upon this class of hospital, and as a matter of fact, although the accommodation is taxed to the utmost, there is always a long list of patients, medically selected, who are waiting to come in. One great need is an extension of the annual subscription list which, relatively to the expenditure (£1,800 to £13,000), is very inadequate. To increase the reliable income is the present chief need of the charity, and then to still further extend its accommodation. Secretary, Mr. B. Burford Rawlings; Matron, Miss L. C. East.

## Figures and Tables

**Figure f1:**